# Temporal trends in peripartum hysterectomy among individuals with a previous cesarean delivery by race/ethnicity in the United States: A population-based cohort study

**DOI:** 10.1371/journal.pone.0304777

**Published:** 2024-05-31

**Authors:** Maya Rajasingham, Parnian Hossein Pour, Sarah Scattolon, Giulia M. Muraca

**Affiliations:** 1 Department of Obstetrics and Gynecology, Faculty of Health Sciences, McMaster University, Hamilton, ON, Canada; 2 Faculty of Health Sciences, Department of Health Research Methods, Evidence and Impact, McMaster University, Hamilton, ON, Canada; 3 Department of Medicine, Solna, Karolinska Institutet, Eugeniahemmet, Clinical Epidemiology Unit, Stockholm, Sweden; Kwame Nkrumah University of Science and Technology College of Health Sciences, GHANA

## Abstract

**Objectives:**

Rates of severe maternal morbidity have highlighted persistent and growing racial disparities in the United States (US). We aimed to contrast temporal trends in peripartum hysterectomy by race/ethnicity and quantify the contribution of changes in maternal and obstetric factors to temporal variations in hysterectomy rates.

**Methods:**

We conducted a population-based, retrospective study of 5,739,569 US residents with a previous cesarean delivery, using National Vital Statistics System’s Natality Files (2011–2021). Individuals were stratified by self-identified race/ethnicity and classified into four periods based on year of delivery. Temporal changes in hysterectomy rates were estimated using odds ratios (ORs) and 95% confidence intervals (CIs). We used sequential logistic regression models to quantify the contribution of maternal and obstetric factors to temporal variations in hysterectomy rates.

**Results:**

Over the study period, the peripartum hysterectomy rate increased from 1.23 (2011–2013) to 1.44 (2019–2021) per 1,000 deliveries (OR 2019–2021 vs. 2011–2013 = 1.17, 95% CI 1.10 to 1.25). Hysterectomy rates varied by race/ethnicity with the highest rates among Native Hawaiian and Other Pacific Islander (NHOPI; 2.73 per 1,000 deliveries) and American Indian or Alaskan Native (AIAN; 2.67 per 1,000 deliveries) populations in 2019–2021. Unadjusted models showed a temporal increase in hysterectomy rates among AIAN (2011–2013 rate = 1.43 per 1,000 deliveries; OR 2019–2021 vs. 2011–2013 = 1.87, 95% CI 1.02 to 3.45) and White (2011–2013 rate = 1.13 per 1,000 deliveries; OR 2019–2021 vs. 2011–2013 = 1.21, 95% CI 1.11 to 1.33) populations. Adjustment ranged from having no effect among NHOPI individuals to explaining 14.0% of the observed 21.0% increase in hysterectomy rates among White individuals.

**Conclusion:**

Nationally, racial disparities in peripartum hysterectomy are evident. Between 2011–2021, the rate of hysterectomy increased; however, this increase was confined to AIAN and White individuals.

## Introduction

In the United States (US), severe maternal morbidity, including acute myocardial infarction, peripartum hysterectomy, and acute renal failure, is of growing concern [1]. Notably, rates of severe maternal morbidity have increased from 1.3% in 2008 to 2.1% in 2021, with race/ethnicity-specific increases of varying magnitudes [2]. An integral component of improving obstetric care involves understanding how secular changes in maternal morbidity determinants relate to temporal variations in rates of specific morbidity outcomes.

One cause of severe maternal morbidity is peripartum hysterectomy, which describes the surgical removal of the uterus during delivery or postpartum. Defined by the World Health Organization [3] as a near-miss event, this intervention is primarily performed to control life-threating obstetric hemorrhage, uterine rupture, abnormally invasive placenta, or sepsis [4–6]. While a peripartum hysterectomy can be lifesaving, it is associated with morbidity (further blood loss, infection) and mortality [7–9]. Peripartum hysterectomy is an important obstetric surveillance measure as it is frequently used in combination with postpartum hemorrhage (PPH) as an indicator of severe PPH. In the event of severe PPH, peripartum hysterectomy is indicated when conservative treatments to control bleeding have failed, such as medical (i.e., uterotonic or antifibrinolytic agents, tamponade techniques, uterine artery embolization) or surgical management (i.e., vascular ligation, uterine compression sutures) [4,10]. There are three main surgical approaches for peripartum hysterectomy: abdominal (most common), vaginal, or laparoscopic [7]. In the US, the rate of hysterectomy increased from 0.87 to 1.28 per 1,000 deliveries between 2008 and 2021 [2]. It remains unknown whether this increase is reflective of equal or disproportionate changes in rates of hysterectomy by race/ethnicity as well as if any potential disparities are narrowing or widening over time.

The rise in peripartum hysterectomy in the US has occurred concomitantly with increasing rates of cesarean delivery and placenta accreta spectrum disorders. Subsequently, the predominant indication for peripartum hysterectomy has shifted from uterine atony to placenta disorders [7,11]. Risk factors for placental disorders include a previous cesarean delivery, with each successive cesarean compounding an individual’s risk [12]. Given this elevated risk, understanding trends of peripartum hysterectomy among individuals with a previous cesarean delivery is particularly valuable given the growth of this population, which has been shown to be differentially distributed among racial/ethnic groups [13–21].

Previous literature has demonstrated an association between race/ethnicity and peripartum hysterectomy [22–25], and evaluated trends over time [2,11,22,26]. However, these studies do not assess potential temporal variation in hysterectomy rates or investigate factors that may contribute to race/ethnicity-specific trends. We sought to compare temporal trends in rates of peripartum hysterectomy by race/ethnicity among US residents with a previous cesarean delivery. Further, we aimed to quantify the influence of maternal and obstetric factors on temporal variations in hysterectomy rates.

## Materials and methods

### Study design

This retrospective cohort study was conducted using the US National Vital Statistics System’s (NVSS) Natality Files. Annually, NVSS collects information on approximately 99% of all registered births within the 50 US states and the District of Columbia [27]. Data is abstracted from birth certificates and representative of the national population. The Natality Files provides information on a variety of measures including individual maternal characteristics (i.e., age, pre-pregnancy body mass index [BMI], and marital status), co-morbidity indicators (i.e., pre-existing and gestational diabetes, and use of assisted reproductive technology), and obstetric practice factors (i.e., mode of delivery, induction of labour, and augmentation of labour) [27]. Additionally, the dataset contains information on maternal and paternal race/ethnicity. This data is self-reported and collected through a Mother’s Worksheet at the time of birth [27]. If a mother’s race is not reported, the race of the father is assigned to the mother. When information on neither parent is available, the race of the mother is imputed based on a previous birth certificate record with a known maternal race, if one exist [27].

This study was restricted to the years of 2011 to 2021 to include all available data on maternal morbidity outcomes within the Natality Files. Between years, coding inconsistencies within variables were rectified by recoding the data to match 2021 encodings.

### Study population

We identified all US residents with a live birth between 2011 and 2021. Individuals were excluded if they did not have a previous cesarean delivery, and/or had missing data on any study covariate except for assisted reproductive technology which had a missingness of 6.9%.

Individuals were stratified by self-reported race/ethnicity based on the following categories: non-Hispanic American Indians or Alaska Natives (AIAN), non-Hispanic Asian, non-Hispanic Black, Hispanic, non-Hispanic Native Hawaiian and Other Pacific Islander (NHOPI), non-Hispanic White, and non-Hispanic more than one race. These race/ethnicity categories will be referred to as AIAN, Asian, Black, Hispanic, NHOPI, White, and more than one race, respectively.

### Primary independent and dependent variables

The independent variable of interest was calendar year of delivery. Individuals were classified by year and by period (2011–2013, 2014–2015, 2016–2018, and 2019–2021). For Asian and NHOPI populations, analyses were restricted to births between 2014 and 2021 (periods; 2014–2015, 2016–2018, 2019–2021) as we were unable to match 2021 encodings of race/ethnicity for these groups between 2011 and 2013.

The outcome of interest (dependent variable) was peripartum hysterectomy, which was defined as the “*surgical removal of the uterus that was not planned before the [birth hospital] admission”* [28].

### Covariates

Clinical characteristics associated with peripartum hysterectomy were identified *a priori* based on previous literature and clinical expertise [4,22,23]. Data on the following covariates were obtained: maternal age (< 19, 19–24, 30–34, 35–40, > 40 vs. 25–29 years), parity (two or more prior deliveries vs. one prior delivery), pre-pregnancy BMI (underweight < 18.5 kg/m^2^, overweight 25.0–29.9 kg/m^2^, obese ≥ 30 kg/m^2^, vs. normal 18.5–24.9 kg/m^2^), number of previous cesarean deliveries (two or more prior cesarean deliveries vs. one prior cesarean delivery), assisted reproductive technology (yes, unknown, vs. no), multiple gestations, pre-pregnancy diabetes, pre-pregnancy hypertension, gestational diabetes, preeclampsia/eclampsia, trial of labour, induction of labour, augmentation of labour, and high infant birth weight (≥ 4,000g vs. < 4,000g).

### Statistical analysis

All analyses were conducted for the overall study cohort and each race/ethnicity, respectively. Since temporal trends in peripartum hysterectomy were linear, the Cochran-Armitage test was used to assess the statistical significance of temporal trends in rates of hysterectomy per 1,000 deliveries by year.

Logistic regression was used to estimate the unadjusted odds ratios (ORs) and 95% confidence intervals (CIs) for peripartum hysterectomy. Temporal changes in peripartum hysterectomy rates were estimated for both year and period using separate regression models. We compared each year/period to the earliest (reference) year/period (i.e., 2021 vs. 2011; 2019–2021 vs. 2011–2013). To quantify the contribution of maternal characteristics and obstetric factors to temporal variations in hysterectomy rates, sequential adjustments were made for individual characteristics (maternal age, parity, pre-pregnancy BMI, number of previous cesarean deliveries, multiple gestations), co-morbidity indicators (assisted reproductive technology, pre-pregnancy diabetes, pre-pregnancy hypertension, gestational diabetes, preeclampsia/eclampsia, high infant birth weight), and obstetric practice factors (trial of labour, induction of labour, augmentation of labour). Sequential adjustments were performed by fitting a series of models with each group of factors being added to the logistic regression in the order described. We compared the unadjusted ORs, adjusted ORs (aORs), and 95% CIs to quantify the contribution of each group of factors on the association between year/period and peripartum hysterectomy.

### Sensitivity analyses

A missing-indicator analysis was conducted where individuals with missing information on assisted reproductive technology were coded as “unknown” and included within our study cohort. Observations with missing, miscoded, or non-reported hysterectomy, race/ethnicity, and/or other covariate data were excluded from the study as they did not exceed 3% of the total study population. To address missing information on assisted reproductive technology, we conducted a sensitivity analysis using a complete-case approach; individuals with missing assisted reproductive technology data were removed from the cohort.

Given that the Natality Files do not contain a patient identifier, we were unable to determine in which prior pregnancy an individual had a cesarean delivery. To address differences in maternal health outcomes based on the number of pregnancies between a cesarean delivery and the index birth, we performed an additional sensitivity analysis restricted to individuals with one prior birth.

We conducted all analyses in RStudio (Version 4.3.0). Statistical significance was set *a priori* at ɑ < 0.05. This study was exempted from ethics approval as all analyses were performed on publicly available data that had been fully anonymized prior to access.

## Results

**Fig 1:**
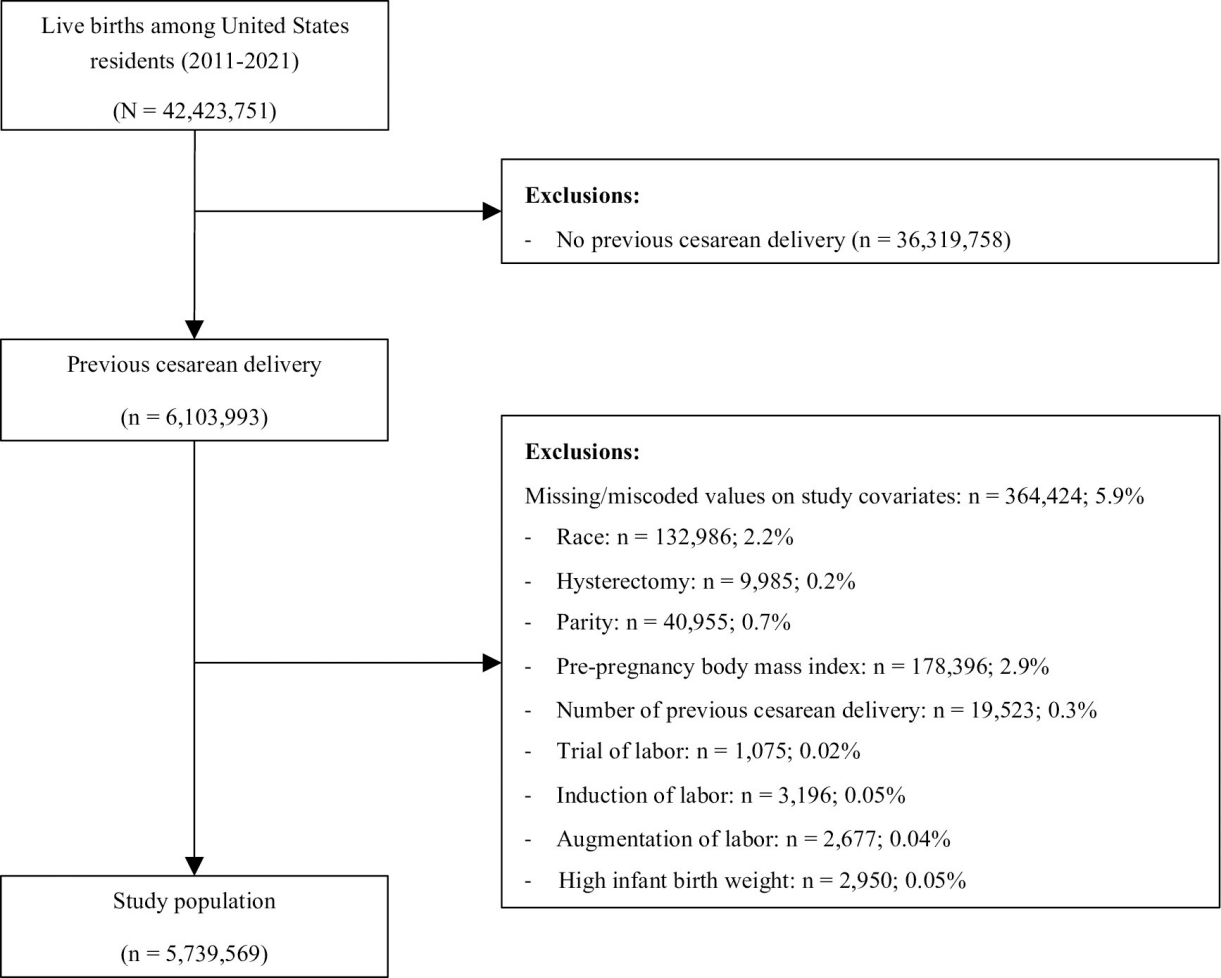
Derivation of study cohort.

Overall, 42,423,751 individuals gave birth between 2011 and 2021 in the US. After exclusions, the study cohort consisted of 5,739,569 individuals who had a previous cesarean delivery (Fig 1). The three largest racial/ethnic groups were White (50.0%), Hispanic (26.4%), and Black individuals (16.0%), respectively (Table 1). Individuals of advanced maternal age comprised of 25.5% of the study population, while 37.5% of individuals were obese and 63.5% had two or more prior deliveries (Table 1). Approximately 1.0% of pregnancies were conceived using assisted reproductive technology, and the proportion of deliveries with a multifetal gestation was 3.7%. The overall rate of peripartum hysterectomy was 1.31 per 1,000 deliveries between 2011 and 2021.

**Table 1:** Demographic and clinical characteristics of individuals with a birth following a previous cesarean delivery, United States, 2011–2021, (N = 5,739,569)^a^.

	2011–2013 (n = 1,288,113)	2014–2015 (n = 1,081,527)	2016–2018 (n = 1,724,551)	2019–2021 (n = 1,645,378)
**Patient demographics**				
Race^b^				
Non-Hispanic AIAN	10,491 (0.8)	9,351 (0.9)	13,655 (0.8)	12,353 (0.8)
Non-Hispanic Asian	-	64,385 (6.0)	109,874 (6.4)	98,892 (6.0)
Non-Hispanic Black	200,314 (15.6)	164,956 (15.3)	277,219 (16.1)	274,664 (16.7)
Hispanic	363,856 (28.2)	276,061 (25.5)	445,415 (25.8)	427,172 (26.0)
Non-Hispanic NHOPI	-	3,003 (0.3)	4,687 (0.3)	4,764 (0.3)
Non-Hispanic White	692,130 (53.7)	544,438 (50.3)	840,628 (48.7)	793,038 (48.2)
Non-Hispanic > 1 race	21,322 (1.7)	19,333 (1.8)	33,073 (1.9)	34,495 (2.1)
Age (years) ^b^				
≤ 19	20,724 (1.6)	11,592 (1.1)	13,548 (0.8)	9,640 (0.6)
20–24	219,227 (17.0)	158,084 (14.6)	208,155 (12.1)	166,607 (10.1)
25–29	370,022 (28.7)	304,550 (28.2)	473,332 (27.4)	425,706 (25.9)
30–34	398,247 (30.9)	351,959 (32.5)	577,241 (33.5)	568,149 (34.5)
35–40	222,303 (17.3)	205,529 (19.0)	364,787 (21.2)	380,007 (23.1)
> 40	57,590 (4.5)	49,813 (4.6)	87,488 (5.1)	95,269 (5.8)
Marital Status				
Married	822,964 (63.9)	698,447 (64.6)	1,013,667 (58.8)	917,494 (55.8)
Unmarried	465,149 (36.1)	383,080 (35.4)	567,281 (32.9)	534,868 (32.5)
Unknown	0 (0)	0 (0)	143,603 (8.3)	193,016 (11.7)
Education				
8^th^ grade or less	65,476 (5.1)	45,259 (4.2)	64,172 (3.7)	56,369 (3.4)
High school, no diploma	167,929 (13.0)	121,423 (11.2)	175,076 (10.2)	145,609 (8.8)
High school graduate or GED	322,662 (25.0)	267,817 (24.8)	438,339 (25.4)	432,378 (26.3)
Some college credit, no degree	277,716 (21.6)	231,750 (21.4)	357,941 (20.8)	327,051 (19.9)
Associate degree	107,180 (8.3)	94,334 (8.7)	151,783 (8.8)	147,121 (8.9)
Bachelor’s degree	218,491 (17.0)	197,705 (18.3)	327,041 (19.0)	322,682 (19.6)
Master’s degree	96,102 (7.5)	90,711 (8.4)	155,347 (9.0)	156,690 (9.5)
Doctorate or professional degree	23,998 (1.9)	24,540 (2.3)	41,621 (2.4)	43,234 (2.6)
Unknown	8,559 (0.6)	7,988 (0.7)	13,231 (0.8)	14,244 (0.9)
Payer				
Medicaid	596,640 (46.3)	495,244 (45.8)	789,065 (45.8)	749,991 (45.6)
Private insurance	576,720 (44.8)	499,596 (46.2)	805,233 (46.7)	781,989 (47.5)
Self-pay	45,404 (3.5)	37,241 (3.4)	60,900 (3.5)	52,892 (3.2)
Other	58,670 (4.6)	43,037 (4.0)	62,364 (3.6)	52,030 (3.2)
Unknown	10,679 (0.8)	6,409 (0.6)	6,989 (0.4)	8,476 (0.5)
Clinical characteristics^b^				
Parity				
1	486,137 (37.7)	407,657 (37.7)	621,354 (36.0)	577,364 (35.1)
≥ 2	801,976 (62.3)	673,870 (62.3)	1,103,197 (64.0)	1,068,014 (64.9)
No. previous cesarean deliveries				
1	897,802 (69.7)	750,207 (69.4)	1,183,187 (68.6)	1,124,374 (68.3)
≥ 2	390,311 (30.3)	331,320 (30.6)	541,364 (31.4)	521,004 (31.7)
Pre-pregnancy BMI kg/m^2^				
Underweight (< 18.5)	26,547 (2.1)	23,204 (2.1)	33,609 (1.9)	26,179 (1.6)
Normal weight (18.5–24.9)	452,390 (35.1)	379,180 (35.1)	574,813 (33.3)	497,140 (30.2)
Overweight (25.0–29.9)	352,791 (27.4)	293,556 (27.1)	470,230 (27.3)	454,925 (27.6)
Obese (≥ 30.0)	456,385 (35.4)	385,587 (35.7)	645,899 (37.5)	667,134 (40.5)
Assisted reproductive technology				
No	1,074,576 (83.4)	992,384 (91.8)	1,645,264 (95.4)	1,583,493 (96.2)
Yes	7,046 (0.5)	7,027 (0.6)	165,93 (1.0)	21,973 (1.3)
Unknown	206,491 (16.0)	82,116 (7.6)	626,94 (3.6)	39,912 (2.4)
Multiple gestation	47,371 (3.7)	39,913 (3.7)	64,991 (3.8)	60,962 (3.7)
Pre-pregnancy diabetes	17,987 (1.4)	15,850 (1.5)	28,634 (1.7)	31,666 (1.9)
Pre-pregnancy hypertension	28,508 (2.2)	26,403 (2.4)	49,752 (2.9)	62,894 (3.8)
Gestational diabetes	86,638 (6.7)	83,466 (7.7)	146,853 (8.5)	168,680 (10.3)
Preeclampsia/eclampsia	60,557 (4.7)	58,340 (5.4)	110,598 (6.4)	134,181 (8.2)
Trial of labour	193,438 (15.0)	176,573 (16.3)	305,681 (17.7)	310,703 (18.9)
Induction of labour	41,000 (3.2)	40,732 (3.8)	78,123 (4.5)	106,113 (6.4)
Augmentation of labour	54,097 (4.2)	53,140 (4.9)	96,941 (5.6)	99,570 (6.1)
High infant birth weight (≥ 4,000 g)	125,887 (9.8)	104,465 (9.7)	163,243 (9.5)	152,964 (9.3)

^a^Values are displayed as column totals and percentages; n (%).

^b^Race, age, and all clinical characteristics had missingness less than 3.0%, except for assisted reproductive technology (6.9%).

AIAN, American Indian or Alaskan Native; > 1 race, more than one race; NHOPI, Native Hawaiian or Other Pacific Islander; GED, general educational development; BMI, body mass index.

Hysterectomy rates varied by race/ethnicity. Within the reference period, rates of hysterectomy were the highest among NHOPI (2.66 per 1,000 deliveries), more than one race (1.50 per 1,000 deliveries), and Black (1.49 per 1,000 deliveries) populations (Table 2). The rate of peripartum hysterectomy significantly increased from 1.08 to 1.47 per 1,000 deliveries (2011 to 2021; *p* for trend < 0.001; S1 Table). A similar trend was noted among White individuals where the rate of peripartum hysterectomy increased from 0.98 to 1.44 per 1,000 deliveries (2011 to 2021; *p* for trend < 0.001; S1 Table). Rates also increased among Black individuals over the study period (1.18 to 1.65 per 1,000 deliveries, percent change = 39.8%, *p* for trend = 0.03). The largest increase in hysterectomy was observed among AIAN individuals from 0.92 to 3.30 per 1,000 deliveries between 2011 and 2021. However, owing to the low frequency of events, this change was not statistically significant (*p* for trend = 0.06; S1 Table). In the most recent year (2021), the highest rates of hysterectomy were among AIAN (3.30 per 1,000 deliveries) and NHOPI (3.19 per 1,000 deliveries) populations (S1 Table).

**Table 2:** Rates of peripartum hysterectomy (per 1,000 deliveries) over time by race/ethnicity in individuals with a previous caesarean delivery, United States, 2011–2021.

	2011–2013	2014–2015	2016–2018	2019–2021	% change*	*p* value^†^
AIAN	1.43	3.21	2.49	2.67	86.7	0.06
Asian	-	1.32	1.16	1.34	1.5	0.49
Black	1.49	1.39	1.48	1.66	11.4	**0.03**
Hispanic	1.25	1.38	1.37	1.39	11.2	0.08
NHOPI	-	2.66	1.49	2.73	2.6	0.69
White	1.13	1.13	1.17	1.37	21.2	**< 0.001**
>1 race	1.50	1.45	1.15	1.42	-5.3	0.83
Overall	1.23	1.27	1.28	1.44	17.1	**< 0.001**

*Percent change in peripartum hysterectomy rate in 2019–2021 vs. 2011–2013.

^†^*p*-value of Cochran-Armitage test for linear trend in hysterectomy rate by year of delivery. Statistical significance was set at α < 0.05.

AIAN, American Indian or Alaskan Native; NHOPI, Native Hawaiian or Other Pacific Islander.; > 1 race, more than one race. All race/ethnicity categories were restricted to non-Hispanic individuals, except for those in the Hispanic group.

**Fig 2:**
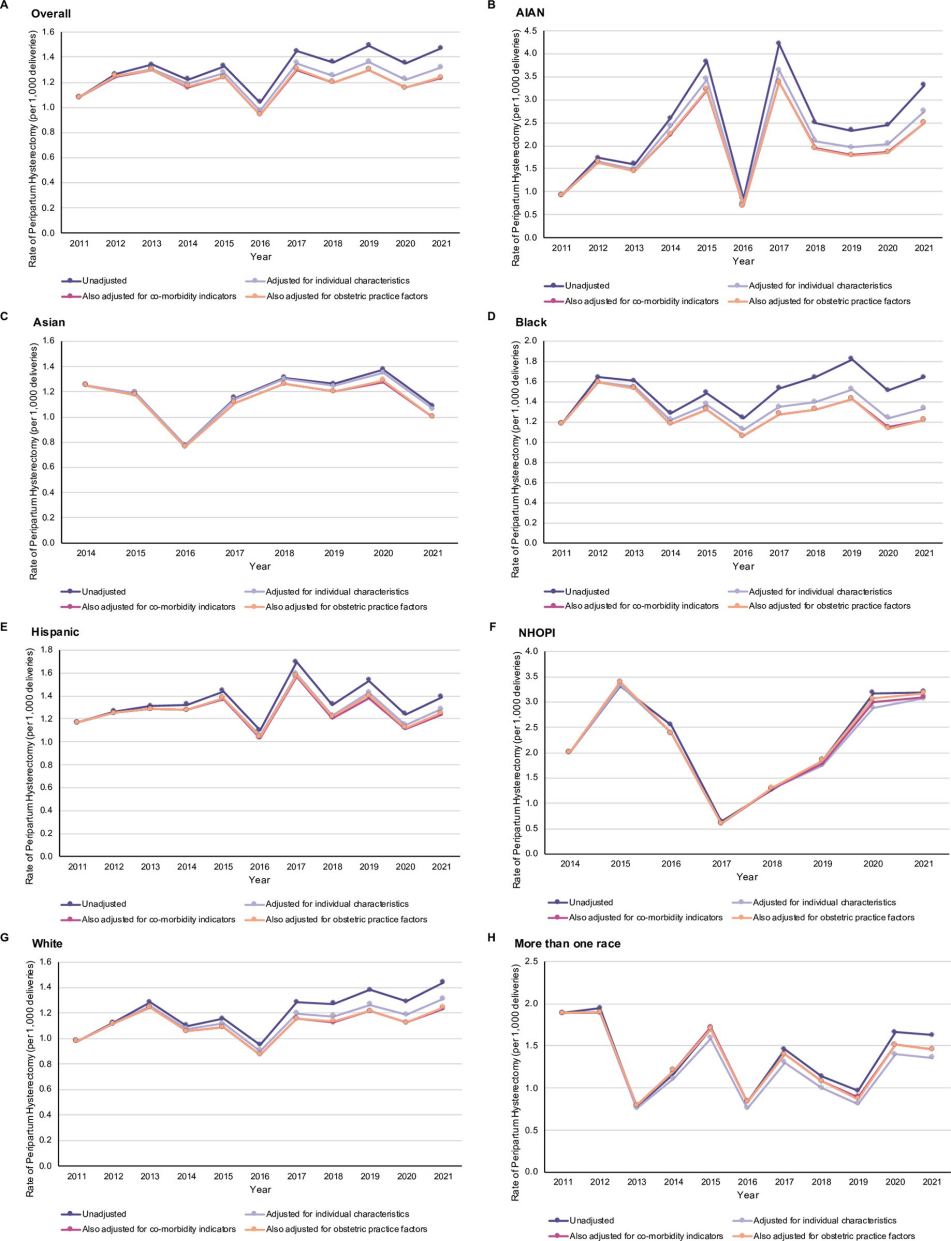
Crude and sequentially adjusted rates of peripartum hysterectomy by race/ethnicity, United States, 2011–2021. Rates are presented for the: (A) overall study cohort, (B) non-Hispanic American Indians or Alaska Natives (AIAN), (C) non-Hispanic Asian, (D) non-Hispanic Black, (E) Hispanic, (F) non-Hispanic Native Hawaiian and Other Pacific Islander (NHOPI), (G) non-Hispanic White, (H) and non-Hispanic more than one race. Note that y-axis scales differ by race/ethnicity.

S1 Fig presents the temporal changes in maternal and obstetric factors for the overall study cohort. The proportion of individuals who were overweight/obese, had more than one previous birth, were of advanced maternal age, had gestational diabetes, preeclampsia/eclampsia, a trial of labour, and who had their labour induced or augmented increased over the study period. Overall, the unadjusted model showed an increased rate of peripartum hysterectomy over time (OR 2019–2021 vs. 2011–2013 = 1.17, 95% CI 1.10 to 1.25, *p* < 0.001; Table 3). Adjustment for individual characteristics accounted for 10% of the observed 17% temporal increase in hysterectomy (Table 3), with maternal age explaining the largest proportion (10%; aOR 2019–2021 vs. 2011–2013 = 1.07, 95% CI 1.00 to 1.14, *p* = 0.04). After sequentially adjusting for co-morbidity indicators and obstetric practice factors, the temporal increase in hysterectomy was entirely explained (aOR 2019–2021 vs 2011–2013 = 1.02, 95% CI 0.95 to 1.08, *p* = 0.65; Fig 2).

**Table 3:** Crude and adjusted odds ratios (ORs) and 95% confidence intervals (CIs) for peripartum hysterectomy in 2019–2021 vs. reference epoch, among individuals with a previous cesarean delivery, United States, 2011–2021.

	Overall OR (95% CI) n = 5,739,569		AIAN OR (95% CI) n = 45,850		Asian OR (95% CI) n = 273,151		Black OR (95% CI) n = 917,153		Hispanic OR (95% CI) n = 1,512,504		NHOPI OR (95% CI) n = 12,454		White OR (95% CI) n = 2,870,234		> 1 race OR (95% CI) n = 108,223
**Unadjusted**																
2019–2021 vs. reference epoch	**1.17** **(1.10, 1.25)**		**1.87** **(1.02, 3.45)**		1.02 (0.78, 1.34)		1.12 (0.96, 1.29)		1.11 (0.98, 1.25)		1.02 (0.42, 2.47)		**1.21** **(1.11, 1.33)**		0.95 (0.61, 1.48)
**Adjusted for individual characteristics (maternal age, parity, pre-pregnancy BMI, number of previous cesarean deliveries, and multiple gestations)**																
2019–2021 vs. reference epoch	1.07 (1.00, 1.14)		1.63 (0.88, 3.01)		1.00 (0.76, 1.32)		0.94 (0.81, 1.08)		1.04 (0.92, 1.18)		0.96 (0.40, 2.33)		**1.11** **(1.02, 1.22)**		0.81 (0.52, 1.27)
**Also adjusted for co-morbidity indicators (assisted reproductive technology, pre-pregnancy diabetes, pre-pregnancy hypertension, gestational diabetes, preeclampsia/eclampsia, and high infant birth weight)**																
2019–2021 vs. reference epoch	1.01 (0.95, 1.08)		1.51 (0.81, 2.81)		0.95 (0.72, 1.26)		0.87 (0.75, 1.01)		1.01 (0.89, 1.14)		0.97 (0.40, 2.36)		1.06 (0.96, 1.17)		0.87 (0.54, 1.38)
**Also adjusted for obstetric practice factors (trial of labour, induction of labour, and augmentation of labour)**															
2019–2021 vs. reference epoch	1.02 (0.95, 1.08)		1.50 (0.81, 2.80)		0.96 (0.73, 1.27)		0.87 (0.75, 1.01)		1.02 (0.90, 1.15)		1.00 (0.41, 2.43)		1.07 (0.97, 1.17)		0.85 (0.54, 1.36)

^a^Reference period for all racial/ethnic groups was 2011–2013, except Asian and NHOPI whose reference epoch was 2014–2015.

Statistical significance was set at α < 0.05. Bolded text indicates statistical significance as per p-values (not shown). Sequential adjustment was performed by fitting a series of models in the order outlined above to quantify the contribution of additional groups of factors on hysterectomy trends over time.

AIAN, American Indian or Alaskan Native; NHOPI, Native Hawaiian or Other Pacific Islander; > 1 race, more than one race. All race/ethnicity categories were restricted to non-Hispanic individuals, except for those in the Hispanic group.

BMI; body mass index.

Temporal changes in individual characteristics, co-morbidity indicators, and obstetric practice factors for each race/ethnicity are included within the (S2 Fig). In summary, the proportion of individuals who were overweight/obese, had more than one previous birth, were of advanced maternal age, and who had a trial of labour increased over time at different magnitudes depending on race/ethnicity. Differences in temporal trends of peripartum hysterectomy were noted by race/ethnicity in both unadjusted and adjusted models when compared to the overall study population. Specifically, no temporal difference in peripartum hysterectomy rates were observed among Asian, Hispanic, NHOPI, and more than one race individuals (Tables 3 and S2). Adjustment did not explain any changes in rates of peripartum hysterectomy over time within NHOPI individuals, while having some effect among Asian, Black, Hispanic, and more than one race populations (Table 3).

Among AIAN individuals, an 87% increase in the rate of peripartum hysterectomy was observed over the study period (OR 2019–2021 vs. 2011–2013 = 1.87, 95% CI 1.02 to 3.45, *p* = 0.04; Table 3). In this population, the observed increase in hysterectomy was entirely explained by adjustment (aOR 2019–2021 vs. 2011–2013 = 1.50, 95% CI 0.81 to 2.80, *p* = 0.20, Fig 2), with the largest proportion being explained by maternal age (22%; aOR 2019–2021 vs. 2011–2013 = 1.65, 95% CI 0.89 to 3.04, *p* = 0.11). Over the study period, the crude hysterectomy rate among White individuals increased from 1.16 to 1.40 per 1,000 deliveries (OR 2019–2021 vs. 2011–2013 = 1.21, 95% CI 1.11 to 1.33, *p* < 0.001; Table 3). In White individuals, adjustment for maternal and obstetric factors explained 14.0% of the observed 21.0% increase, with maternal age providing the largest explanation (8%; OR 2019–2021 vs. 2011–2013 = 1.13, 95% CI 1.03 to 1.24, *p* = 0.01; Table 3). However, after all adjustments, the increasing peripartum hysterectomy rate among this population was no longer statistically significant (aOR 2019–2021 vs. 2011–2013 = 1.07, 95% CI 0.97 to 1.17, *p* = 0.18).

### Sensitivity analyses results

Models with a missing indicator for assisted reproductive technology (n = 5,739,569) and models restricted to individuals with complete information (n = 5,348,356) yielded comparable rates of peripartum hysterectomy over time across all racial/ethnic populations except among AIAN individuals (S3 Table). Among AIAN individuals, no temporal differences in peripartum hysterectomy rates were observed (2011–2013 rate = 1.46 per 1,000 deliveries; OR 2019–2021 vs. 2011–2013 = 1.84, 95% CI 0.98 to 3.44, *p* = 0.06).

After restricting to individuals with a previous cesarean delivery and one prior birth (n = 1,947,389), the overall rate of peripartum hysterectomy across all years was 0.59 per 1,000 deliveries. There were two main differences between the restricted population and all individuals with a previous cesarean delivery. First, a greater relative increase in hysterectomy rates over time was observed among the restricted population. In the overall population of individuals with one prior birth, the rate of hysterectomy increased by 38% between 2011–2013 and 2019–2021 (2011–2013 rate = 0.47 per 1,000 deliveries; OR 2019–2021 vs. 2011–2013 = 1.38, 95% CI 1.16 to 1.65, *p* < 0.001; S4 Table), and by 49% in White individuals with one prior birth over the same period (2011–2013 rate = 0.45 per 1,000 deliveries; OR 2019–2021 vs. 2011–2013 = 1.49, 95% CI 1.17 to 1.90, *p* = 0.01; S4 Table). Second, after adjustment, the temporal increase in hysterectomy rates remained statistically significant across all periods among the overall and White restricted populations (S4 Table).

## Discussion

We conducted a population-based cohort study of 5,739,569 deliveries in the US between 2011 and 2021 to assess temporal variations in peripartum hysterectomy rates by race/ethnicity with and without adjustment for maternal characteristics and obstetric factors. Over the study period, the rate of peripartum hysterectomy increased by 17.1% (1.23 per 1,000 deliveries to 1.44 per 1,000 deliveries). However, this increase was not observed across all racial/ethnic groups; it was confined to AIAN, Black and White populations. Adjustment for individual characteristics, co-morbidity indicators, and obstetric practice factors ranged from having no effect among NHOPI individuals to explaining 14% of the 21% increase in hysterectomy rates among White individuals. Across all racial/ethnic groups and the overall study population, no temporal differences (2019–2021 vs. 2011–2013) in hysterectomy rates were observed after adjustment. This suggests that the observed temporal increase in hysterectomy rates between 2011 and 2021 can be explained by changes in maternal and obstetric practices factors that occurred over this period. Nonetheless, of concern are the consistently high rates of peripartum hysterectomy among AIAN and NHOPI individuals in comparison to other racial/ethnic groups.

The evaluation of racial health disparities is challenging as race is a social construct that is used as a proxy measure for the effects of discrimination and systemic marginalization. These include differences in the rates of maternal health indicators by race, such as cesarean delivery and co-morbid conditions, which are known risk factors for severe maternal morbidity [2]. Over time, the prevalence of these risk factors have increased at varying magnitudes across racial/ethnic populations [15,16,29,30]. While there is a known association between race/ethnicity and peripartum hysterectomy [22–25], this analysis adds to the literature by considering how changes in maternal health indicators within racial/ethnic populations have contributed to disparities.

To our knowledge, no study has evaluated temporal trends of peripartum hysterectomy stratified by race/ethnicity among a high-risk population (i.e., individuals with a previous cesarean delivery). The few studies that have included race/ethnicity in their analyses have done so to quantify its relationship with peripartum hysterectomy and not to analyze trends over time or the contribution of maternal health indicators to race/ethnicity-specific rates. In agreement with our study, research has noted higher rates of peripartum hysterectomy among racial/ethnic populations in comparison to White individuals [22,24]. Specifically, higher hysterectomy rates were found among Asian (1.20 per 1,000), Black (1.11 per 10,000), and Hispanic (1.05 per 1,000) populations compared to White individuals (0.76 per 1,000) [22]. However, studies evaluating rates of hysterectomy over time have reported mixed results. Similar to our findings, population-based studies in the US have found temporal increases in the rate of hysterectomy between 1994 to 2007 [31], 2004 to 2014 [22], and 2008 to 2021 [2]. Another population-based study found no significant increase in rates from 1998 to 2003, although this may be explained by the increasing number of states who contributed to the Healthcare Cost and Utilization project over the study period [32].

Strengths of our study include an analysis of a large number of deliveries in the US given the use of a nationally representative, population-based dataset. Data was also consistently collected over the study period allowing for a robust analysis of a rare outcome over time. Further, to not mask any temporal variations in hysterectomy rates by race/ethnicity, our analyses did not adjust for clinical indications of hysterectomy which vary by race/ethnicity.

The limitations of our study include low sensitivity of the peripartum hysterectomy indicator within birth certificate data and the staggered adoption of the 2003 birth certificate across states until 2016 [27,33]. With that said, we do not expect the hysterectomy indicator to be differentially insensitive by race/ethnicity minimally biasing our comparative results. Another limitation of this study is the sharp decrease in hysterectomy rates observed in 2016, which is inconsistent with the temporal trends preceding and following this year across all races/ethnicities. A *post hoc*, state level analysis in CDC Wonder [34] was conducted to explore possible reasons for this drop in rates given the staggered adoption of the 2003 birth certificate. However, we were not able to develop a plausible explanation. A 2023 US-based study by Fink et al., [2] which used the Premier PINC AI Healthcare database, noted a similar sharp decrease in rates of severe maternal morbidity in 2016; though after adjustment for confounders, the decrease was entirely explained. It is worth noting that Fink et al. [2] did investigate a different population (all individuals with a birth between 2008 and 2021) than the present study as well as include confounders which were not available in our dataset. This may explain why we observed persistent sharp decreases in rates of hysterectomy during 2016 after adjustment. Given that our data source did not contain a patient identifier, we were unable to determine in which prior pregnancy an individual had a cesarean delivery. To explore differences in hysterectomy risk based on the number of elapsed deliveries between a previous cesarean and index birth, we conducted a sensitivity analysis restricted to individuals with one prior birth. In this analysis, we found a temporal increases in peripartum hysterectomy rates across all periods when compared to the results from our main analyses. Future studies are needed to explore the potential mediating effect of the number of pregnancies between a cesarean delivery and index birth on the risk of peripartum hysterectomy. Additionally, 6.9% of individuals were missing data on the use of assisted reproductive technology, which we included in our main models using a missing indicator. Results from our sensitivity analyses including individuals with complete information were comparable to our main findings, except for no observed temporal increase in hysterectomy rates among AIAN individuals. However, given that we do not know if the frequency of assisted reproductive technology use differed between those with missing and available information, it is difficult to speculate on the magnitude and direction of bias introduced by missing data on this variable. Finally, we were not able to account for important obstetric history details, such as an individual having multiple pregnancies over the study period, as this information was not available in our data source.

We explored the crude and adjusted temporal trend of peripartum hysterectomy by race/ethnicity among individuals with a previous caesarean delivery. Between 2011 and 2021, the rate of hysterectomy increased; however, this increase was confined to AIAN and White individuals. We must note that this study may not reflect temporal variations in peripartum hysterectomy across all deliveries in the US, given that individuals with a previous caesarean delivery comprised of 14.4% of all births between 2011 and 2021. However, the high rate of placental disorders in individuals with a previous cesarean delivery largely contributes to the overall peripartum hysterectomy rate.

## Conclusion

Racial disparities in rates of hysterectomy are evident in the US with the highest rates among AIAN and NHOPI individuals. Rates of peripartum hysterectomy increased from 2011–2021 among AIAN and White populations. Maternal characteristics, co-morbidity indicators, and obstetric practice factors differentially explain the temporal increase in peripartum hysterectomy rates by race/ethnicity. Clinicians and public health officials must consider the historic and structural reasons behind elevated peripartum hysterectomy rates among racial/ethnic groups. This understanding is important for developing targeted interventions that narrow, rather than widen, maternal health inequities.

## Supporting information

S1 Fig Temporal trends of individual characteristics, co-morbidity indicators, and obstetric practice factors among the study cohort, United states, 2011–2021. In those with a previous cesarean delivery, the precent of deliveries with peripartum hysterectomy determinants.
(PDF)

S2 Fig Temporal trends of individual characteristics, co-morbidity indicators, and obstetric practice factors by race/ethnicity, United states, 2011–2021. Precent of deliveries with peripartum hysterectomy determinants stratified by each race/ethnicity.
(PDF)

S1 Table Rates of peripartum hysterectomy (per 1,000 individuals) over time by race/ethnicity in individuals with a previous caesarean delivery, United States, 2011–2021. Race/ethnicity-specific hysterectomy rates by year, percent change in hysterectomy rates, and p-value for linear trend over the study period.
(DOCX)

S2 Table Crude and adjusted odds ratios (ORs) and 95% confidence intervals (CIs) for peripartum hysterectomy by epoch, among individuals with a previous cesarean delivery and complete information (complete case analysis), United States, 2011–2021. Complete case analysis of temporal changes in hysterectomy rates by race/ethnicity.
(DOCX)

S3 Table Crude and adjusted odds ratios (ORs) and 95% confidence intervals (CIs) for peripartum hysterectomy by epoch, among individuals with a previous cesarean delivery and missing information on assisted reproductive technology (sensitivity analysis), United States, 2011–2021. Results from analyses included deliveries missing on assisted reproductive technology using a missing-indicator approach.
(DOCX)

S4 Table Crude and adjusted odds ratios (ORs) and 95% confidence intervals (CIs) for peripartum hysterectomy by epoch, among individuals with a previous cesarean delivery and one prior childbirth (sensitivity analysis), United States, 2011–2021. Analysis of primiparous individuals to investigate heterogeneity of the study cohort.
(DOCX)

## Abbreviations

aOR: Adjusted odds ratio
AIAN: America Indian or Alaskan Native
BMI: Body mass index
CDC: Centers for Disease Control and Prevention
CI: Confidence interval
NHOPI: Native Hawaiian and Other Pacific Islander. Islander
NVSS: National Vital Statistics System
OR: Odds ratio
PPH: Postpartum hemorrhage
US: United States
