# Supplementary material for: Temporal trends in peripartum hysterectomy among individuals with a previous cesarean delivery by race/ethnicity in the United States: A population-based cohort study
Source: PLoS One. 2024 May 31;19(5):e0304777. doi: 10.1371/journal.pone.0304777 (PMC11142665; doi:10.1371/journal.pone.0304777)

S1 Fig: Temporal trends of individual characteristics, co-morbidity indicators, and obstetric practice factors among the study cohort, United States, 2011–2021.

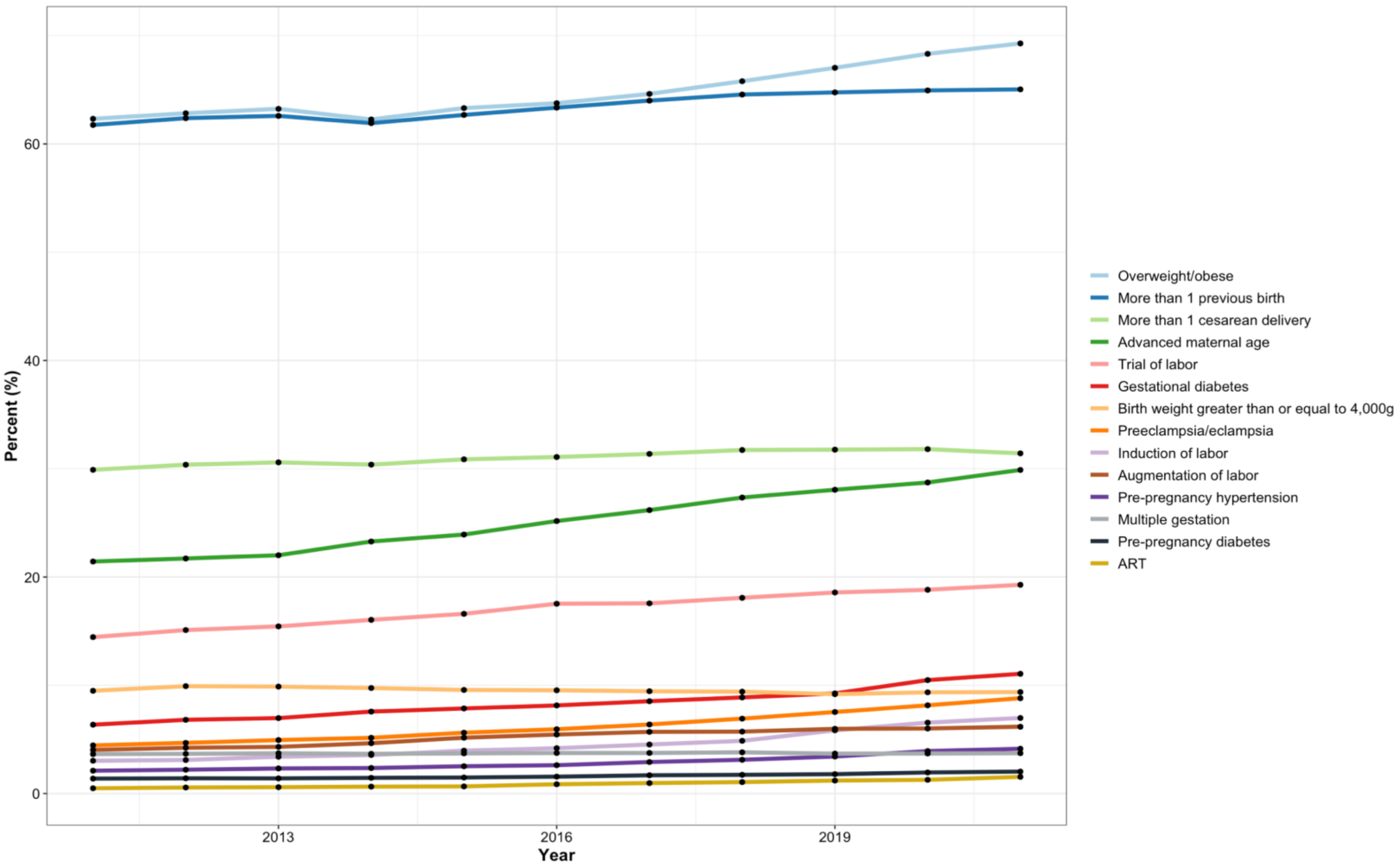

Supplement: S1 Fig — In those with a previous cesarean delivery, the precent of deliveries with peripartum hysterectomy determinants. (PDF) [file pone.0304777.s001.pdf]
