# Supplementary material for: Temporal trends in peripartum hysterectomy among individuals with a previous cesarean delivery by race/ethnicity in the United States: A population-based cohort study
Source: PLoS One. 2024 May 31;19(5):e0304777. doi: 10.1371/journal.pone.0304777 (PMC11142665; doi:10.1371/journal.pone.0304777)
Supplement: S1 Table — Race/ethnicity-specific hysterectomy rates by year, percent change in hysterectomy rates, and p-value for linear trend over the study period. (DOCX) [file pone.0304777.s003.docx]

S1 Table: Rates of peripartum hysterectomy (per 1,000 individuals) by race/ethnicity in individuals with a previous caesarean delivery, United States, 2011-2021

|  | 2011 | 2012 | 2013 | 2014 | 2015 | 2016 | 2017 | 2018 | 2019 | 2020 | 2021 | % change^*^ | | *p* value^†^ |
| --- | --- | --- | --- | --- | --- | --- | --- | --- | --- | --- | --- | --- | --- | --- |
| AIAN | 0.92 | 1.72 | 1.60 | 2.59 | 3.81 | 0.85 | 4.20 | 2.49 | 2.32 | 2.44 | 3.30 | 258.7 | 0.06 | |
| Asian | - | - | - | 1.35 | 1.29 | 0.84 | 1.24 | 1.42 | 1.37 | 1.49 | 1.17 | -13.3 | 0.49 | |
| Black | 1.18 | 1.64 | 1.60 | 1.29 | 1.48 | 1.24 | 1.53 | 1.64 | 1.82 | 1.51 | 1.65 | 39.8 | **0.03** | |
| Hispanic | 1.17 | 1.26 | 1.31 | 1.32 | 1.44 | 1.10 | 1.69 | 1.32 | 1.53 | 1.24 | 1.39 | 18.8 | 0.08 | |
| NHOPI | - | - | - | 2.01 | 3.32 | 2.55 | 0.65 | 1.27 | 1.85 | 3.16 | 3.19 | 37.0 | 0.69 | |
| White | 0.98 | 1.13 | 1.28 | 1.10 | 1.15 | 0.95 | 1.28 | 1.27 | 1.38 | 1.29 | 1.44 | 46.9 | **< 0.001** | |
| > 1 race | 1.89 | 1.95 | 0.77 | 1.18 | 1.70 | 0.84 | 1.46 | 1.14 | 0.96 | 1.67 | 1.63 | -13.8 | 0.83 | |
| Overall | 1.08 | 1.26 | 1.33 | 1.22 | 1.33 | 1.03 | 1.45 | 1.36 | 1.49 | 1.35 | 1.47 | 36.1 | **< 0.001** | |

^*^Percent change in peripartum hysterectomy rate in 2021 vs. 2011.

^†^*p*-value of Cochran-Armitage test for linear trend in hysterectomy rate by year of delivery. Statistical significance was set at α < 0.05.

AIAN, American Indian or Alaskan Native; NHOPI, Native Hawaiian or Other Pacific Islander; > 1 race, more than one race. All race/ethnicity categories were restricted to non-Hispanic individuals, except for those in the Hispanic group.
