# Supplementary material for: Temporal trends in peripartum hysterectomy among individuals with a previous cesarean delivery by race/ethnicity in the United States: A population-based cohort study
Source: PLoS One. 2024 May 31;19(5):e0304777. doi: 10.1371/journal.pone.0304777 (PMC11142665; doi:10.1371/journal.pone.0304777)
Supplement: S2 Table — Complete case analysis of temporal changes in hysterectomy rates by race/ethnicity. (DOCX) [file pone.0304777.s004.docx]

S2 Table. Crude and adjusted odds ratios (ORs) and 95% confidence intervals (CIs) for peripartum hysterectomy by period, among individuals with a previous cesarean delivery, United States, 2011-2021.

|  | Overall  n=5,739,569 | | AIAN  n=45,850 | | Asian  n=273,151 | | Black  n=917,153 | | Hispanic  n=1,512,504 | | NHOPI  n=12,454 | | White  n=2,870,234 | | > 1 race  n=108,223 |  |
| --- | --- | --- | --- | --- | --- | --- | --- | --- | --- | --- | --- | --- | --- | --- | --- | --- |
| Hysterectomy per 1,000 deliveries in reference period | 1.23 | | 1.43 | | 1.32 | | 1.49 | | 1.25 | | 2.66 | | 1.13 | | 1.50 |  |
|  | **OR (95% CI)** | | **OR (95% CI)** | | **OR (95% CI)** | | **OR (95% CI)** | | **OR (95% CI)** | | **OR (95% CI)** | | **OR (95% CI)** | | **OR (95% CI)** |  |
| Unadjusted | |  | |  | |  | |  | |  | |  | |  | | |
| 2011-2013 | Ref | | Ref | | - | | Ref | | Ref | | - | | Ref | | Ref |  |
| 2014-2015 | 1.03  (0.96, 1.11) | | **2.25**  **(1.21, 4.18)** | | Ref | | 0.93  (0.79, 1.11) | | 1.11  (0.97, 1.27) | | Ref | | 0.99  (0.89, 1.11) | | 0.96  (0.58, 1.60) |  |
| 2016-2018 | 1.04  (0.98, 1.11) | | 1.74  (0.95, 3.20) | | 0.88  (0.67, 1.16) | | 0.99  (0.85, 1.15) | | 1.10  (0.97, 1.24) | | 0.56  (0.20, 1.55) | | 1.03  (0.94, 1.13) | | 0.77  (0.48, 1.23) |  |
| 2019-2021 | **1.17**  **(1.10, 1.25)** | | **1.87**  **(1.02, 3.45)** | | 1.02  (0.78, 1.34) | | 1.12  (0.96, 1.29) | | 1.11  (0.98, 1.25) | | 1.02  (0.42, 2.47) | | **1.21**  **(1.11, 1.33)** | | 0.95  (0.61, 1.48) |  |
| Adjusted for individual characteristics (maternal age, parity, pre-pregnancy BMI, number of previous cesarean deliveries, and multiple gestations) | | | | | | | | | | | | | | | | |
| 2011-2013 | Ref | | Ref | | - | | Ref | | Ref | | - | | Ref | | Ref |  |
| 2014-2015 | 1.01  (0.94, 1.08) | | **2.14**  **(1.15, 3.98)** | | Ref | | 0.89  (0.75, 1.06) | | 1.08  (0.94, 1.24) | | Ref | | 0.97  (0.88, 1.08) | | 0.92  (0.56, 1.54) |  |
| 2016-2018 | 0.98  (0.92, 1.04) | | 1.56  (0.85, 2.88) | | 0.88  (0.67, 1.16) | | 0.89  (0.76, 1.03) | | 1.04  (0.92, 1.18) | | 0.54  (0.19, 1.48) | | 0.97  (0.88, 1.06) | | 0.70  (0.43, 1.11) |  |
| 2019-2021 | 1.07  (1.00, 1.14) | | 1.63  (0.88, 3.01) | | 1.00  (0.76, 1.32) | | 0.94  (0.81, 1.08) | | 1.04  (0.92, 1.18) | | 0.96  (0.40, 2.33) | | **1.11**  **(1.02, 1.22)** | | 0.81  (0.52, 1.27) |  |
| Also adjusted for co-morbidity indicators (assisted reproductive technology, pre-pregnancy diabetes, pre-pregnancy hypertension, gestational diabetes, preeclampsia/eclampsia, and high infant birth weight) | | | | | | | | | | | | | | | | |
| 2011-2013 | Ref | | Ref | | - | | Ref | | Ref | | - | | Ref | | Ref |  |
| 2014-2015 | 0.99  (0.92, 1.06) | | **2.02**  **(1.08, 3.78)** | | Ref | | 0.87  (0.73, 1.03) | | 1.07  (0.93, 1.23) | | Ref | | 0.96  (0.86, 1.06) | | 0.99  (0.59, 1.65) |  |
| 2016-2018 | 0.94  (0.88, 1.01) | | 1.47  (0.79, 2.72) | | 0.86  (0.65, 1.13) | | 0.84  (0.73, 0.98) | | 1.03  (0.91, 1.16) | | 0.53  (0.19, 1.47) | | 0.94  (0.85, 1.03) | | 0.75  (0.46, 1.22) |  |
| 2019-2021 | 1.01  (0.95, 1.08) | | 1.51  (0.81, 2.81) | | 0.95  (0.72, 1.26) | | 0.87  (0.75, 1.01) | | 1.01  (0.89, 1.14) | | 0.97  (0.40, 2.36) | | 1.06  (0.96, 1.17) | | 0.87  (0.54, 1.38) |  |
| Also adjusted for obstetric practice factors (trial of labour, induction of labour, and augmentation of labour) | | | | | | | | | | | | | | | |  |
| 2011-2013 | Ref | | Ref | | - | | Ref | | Ref | | - | | Ref | | Ref |  |
| 2014-2015 | 0.99  (0.92, 1.07) | | **2.03**  **(1.08, 3.80)** | | Ref | | 0.87  (0.73, 1.03) | | 1.07  (0.94, 1.23) | | Ref | | 0.96  (0.86, 1.07) | | 0.98  (0.59, 1.65) |  |
| 2016-2018 | 0.95  (0.89, 1.01) | | 1.47  (0.79, 2.72) | | 0.86  (0.65, 1.13) | | **0.84**  **(0.73, 0.98)** | | 1.03  (0.91, 1.17) | | 0.53  (0.19, 1.48) | | 0.94  (0.85, 1.04) | | 0.74  (0.46, 1.21) |  |
| 2019-2021 | 1.02  (0.95, 1.08) | | 1.50  (0.81, 2.80) | | 0.96  (0.73, 1.27) | | 0.87  (0.75, 1.01) | | 1.02  (0.90, 1.15) | | 1.00  (0.41, 2.43) | | 1.07  (0.97, 1.17) | | 0.85  (0.54, 1.36) |  |

Statistical significance was set at α < 0.05. Bolded text indicates statistical significance as per p-values (not shown). Sequential adjustment was performed by fitting a series of models in the order outlined above to quantify the contribution of additional groups of factors on hysterectomy trends over time.

AIAN, American Indian or Alaskan Native; NHOPI, Native Hawaiian or Other Pacific Islander; > 1 race, more than one race. All race/ethnicity categories were restricted to non-Hispanic individuals, except for those in the Hispanic group.

BMI; body mass index.
