# Supplementary material for: Temporal trends in peripartum hysterectomy among individuals with a previous cesarean delivery by race/ethnicity in the United States: A population-based cohort study
Source: PLoS One. 2024 May 31;19(5):e0304777. doi: 10.1371/journal.pone.0304777 (PMC11142665; doi:10.1371/journal.pone.0304777)
Supplement: S3 Table — Results from analyses included deliveries missing on assisted reproductive technology using a missing-indicator approach. (DOCX) [file pone.0304777.s005.docx]

S3 Table. Crude and adjusted odds ratios (ORs) and 95% confidence intervals (CIs) for peripartum hysterectomy by period, among individuals with a previous cesarean delivery and complete information (sensitivity analysis), United States, 2011-2021.

|  | Overall  n=5,348,356 | | AIAN  n=44,644 | | Asian  n=268,343 | | Black  n=839,866 | | Hispanic  n=1,470,996 | | NHOPI  n=12,291 | | White  n=2,611,586 | | > 1 race  n=100,630 |  |
| --- | --- | --- | --- | --- | --- | --- | --- | --- | --- | --- | --- | --- | --- | --- | --- | --- |
| Hysterectomy per 1,000 deliveries in reference period | 1.24 | | 1.46 | | 1.31 | | 1.53 | | 1.24 | | 2.72 | | 1.15 | | 1.28 |  |
|  | **OR (95% CI)** | | **OR (95% CI)** | | **OR (95% CI)** | | **OR (95% CI)** | | **OR (95% CI)** | | **OR (95% CI)** | | **OR (95% CI)** | | **OR (95% CI)** |  |
| Unadjusted | |  | |  | |  | |  | |  | |  | |  | | |
| 2011-2013 | Ref | | Ref | | - | | Ref | | Ref | | - | | Ref | | Ref |  |
| 2014-2015 | 1.04  (0.96, 1.12) | | **2.22**  **(1.18, 4.19)** | | Ref | | 0.94  (0.79, 1.13) | | 1.11  (0.97, 1.28) | | Ref | | 0.99  (0.88, 1.11) | | 1.14  (0.65, 2.02) |  |
| 2016-2018 | 1.05  (0.98, 1.12) | | 1.72  (0.92, 3.20) | | 0.89  (0.67, 1.17) | | 1.00  (0.85, 1.17) | | 1.11  (0.98, 1.26) | | 0.56  (0.20, 1.53) | | 1.03  (0.93, 1.14) | | 0.86  (0.50, 1.46) |  |
| 2019-2021 | **1.16**  **(1.09, 1.25)** | | 1.84  (0.98, 3.44) | | 1.04  (0.79, 1.37) | | 1.12  (0.96, 1.30) | | 1.12  (0.99, 1.27) | | 1.01  (0.42, 2.45) | | **1.19**  **(1.08, 1.32)** | | 1.14  (0.69, 1.88) |  |
| Adjusted for individual characteristics (maternal age, parity, pre-pregnancy BMI, number of previous cesarean deliveries, and multiple gestations) | | | | | | | | | | | | | | | | |
| 2011-2013 | Ref | | Ref | | - | | Ref | | Ref | | - | | Ref | | Ref |  |
| 2014-2015 | 1.02  (0.94, 1.10) | | **2.09**  **(1.11, 3.95)** | | Ref | | 0.90  (0.75, 1.08) | | 1.09  (0.94, 1.25) | | Ref | | 0.97  (0.87, 1.09) | | 1.10  (0.62, 1.94) |  |
| 2016-2018 | 0.99  (0.92, 1.06) | | 1.53  (0.82, 2.85) | | 0.88  (0.67, 1.17) | | 0.90  (0.77, 1.05) | | 1.06  (0.94, 1.20) | | 0.53  (0.19, 1.47) | | 0.97  (0.88, 1.08) | | 0.79  (0.46, 1.35) |  |
| 2019-2021 | 1.07  (1.00, 1.14) | | 1.58  (0.84, 2.97) | | 1.02  (0.77, 1.35) | | 0.94  (0.81, 1.10) | | 1.05  (0.93, 1.19) | | 0.95  (0.39, 2.31) | | **1.10**  **(1.00, 1.22)** | | 0.99  (0.60, 1.65) |  |
| Also adjusted for co-morbidity indicators (assisted reproductive technology, pre-pregnancy diabetes, pre-pregnancy hypertension, gestational diabetes, preeclampsia/eclampsia, and high infant birth weight) | | | | | | | | | | | | | | | | |
| 2011-2013 | Ref | | Ref | | - | | Ref | | Ref | | - | | Ref | | Ref |  |
| 2014-2015 | 1.01  (0.94, 1.09) | | **2.07**  **(1.10, 3.91)** | | Ref | | 0.90  (0.75, 1.08) | | 1.08  (0.94, 1.24) | | Ref | | 0.97  (0.87, 1.09) | | 1.11  (0.63, 1.96) |  |
| 2016-2018 | 0.97  (0.91, 1.04) | | 1.50  (0.80, 2.81) | | 0.86  (0.65, 1.14) | | 0.89  (0.76, 1.04) | | 1.04  (0.92, 1.18) | | 0.53  (0.19, 1.47) | | 0.96  (0.87, 1.07) | | 0.78  (0.46, 1.34) |  |
| 2019-2021 | 1.04  (0.97, 1.11) | | 1.54  (0.82, 2.89) | | 0.97  (0.73 ,1.28) | | 0.92  (0.79, 1.07) | | 1.02  (0.90, 1.15) | | 0.97  (0.40, 2.36) | | 1.08  (0.98, 1.19) | | 0.97  (0.59, 1.62) |  |
| Also adjusted for obstetric practice factors (trial of labour, induction of labour, augmentation of labour) | | | | | | | | | | | | | | | |  |
| 2011-2013 | Ref | | Ref | | - | | Ref | | Ref | | - | | Ref | | Ref |  |
| 2014-2015 | 1.01  (0.94, 1.09) | | **2.08**  **(1.10, 3.93)** | | Ref | | 0.90  (0.75, 1.08) | | 1.08  (0.94, 1.24) | | Ref | | 0.97  (0.87, 1.09) | | 1.11  (0.63, 1.96) |  |
| 2016-2018 | 0.98  (0.91, 1.04) | | 1.50  (0.80, 2.81) | | 0.87  (0.65, 1.14) | | 0.89  (0.76, 1.04) | | 1.05  (0.93, 1.19) | | 0.53  (0.19, 1.48) | | 0.97  (0.87, 1.07) | | 0.78  (0.46, 1.33) |  |
| 2019-2021 | 1.04  (0.97, 1.11) | | 1.53  (0.81, 2.88) | | 0.98  (0.74, 1.30) | | 0.92  (0.78, 1.07) | | 1.03  (0.91, 1.16) | | 1.00  (0.41, 2.43) | | 1.08  (0.98, 1.20) | | 0.96  (0.58, 1.60) |  |

Statistical significance was set at α < 0.05. Bolded text indicates statistical significance as per p-values (not shown). Sequential adjustment was performed by fitting a series of models in the order outlined above to quantify the contribution of additional groups of factors on hysterectomy trends over time.

AIAN, American Indian or Alaskan Native; NHOPI, Native Hawaiian or Other Pacific Islander; > 1 race, more than one race. All race/ethnicity categories were restricted to non-Hispanic individuals, except for those in the Hispanic group.

BMI; body mass index.
