# Supplementary material for: Temporal trends in peripartum hysterectomy among individuals with a previous cesarean delivery by race/ethnicity in the United States: A population-based cohort study
Source: PLoS One. 2024 May 31;19(5):e0304777. doi: 10.1371/journal.pone.0304777 (PMC11142665; doi:10.1371/journal.pone.0304777)
Supplement: S4 Table — Analysis of primiparous individuals to investigate heterogeneity of the study cohort. (DOCX) [file pone.0304777.s006.docx]

S4 Table. Crude and adjusted odds ratios (ORs) and 95% confidence intervals (CIs) for peripartum hysterectomy by period, among individuals with a previous cesarean delivery and one prior childbirth (sensitivity analysis), United States, 2011-2021.

|  | Overall  n=1,947,389 | | AIAN  n=11,835 | | Asian  n=139,299 | | Black  n=244.047 | | Hispanic  n=502,646 | | NHOPI  n=3,190 | | White  n=1.012,734 | | > 1 race  n=33,638 |  |
| --- | --- | --- | --- | --- | --- | --- | --- | --- | --- | --- | --- | --- | --- | --- | --- | --- |
| Hysterectomy per 1,000 deliveries in reference period | 0.47 | | 0.71 | | 0.81 | | 0.55 | | 0.44 | | 1.21 | | 0.45 | | 0.47 |  |
|  | **OR (95% CI)** | | **OR (95% CI)** | | **OR (95% CI)** | | **OR (95% CI)** | | **OR (95% CI)** | | **OR (95% CI)** | | **OR (95% CI)** | | **OR (95% CI)** |  |
| Unadjusted | |  | |  | |  | |  | |  | |  | |  | | |
| 2011-2013 | Ref | | Ref | | - | | Ref | | Ref | | - | | Ref | | Ref |  |
| 2014-2015 | **1.32**  **(1.09, 1.60)** | | 0  (0, INF) | | Ref | | 1.12  (0.67, 1.89) | | 1.33  (0.92, 1.04) | | Ref | | **1.31**  **(1.00, 1.71)** | | 1.34  (0.30, 5.98) |  |
| 2016-2018 | **1.33**  **(1.12, 1.59)** | | 1.23  (0.20, 7.35) | | 0.98  (0.61, 1.58) | | 0.83  (0.50, 1.36) | | 1.24  (0.88, 1.75) | | 0  (0, INF) | | **1.45**  **(1.15, 1.85)** | | 1.42  (0.37, 5.49) |  |
| 2019-2021 | **1.38 (1.16, 1.65)** | | 0.46  (0.04, 5.07) | | 1.07  (0.66, 1.74) | | 1.26  (0.79, 1.99) | | 1.04  (0.72, 1.49) | | 1.33  (0.12, 14.75) | | **1.49**  **(1.17, 1.90)** | | 1.58  (0.42, 5.97) |  |
| Adjusted for individual characteristics (maternal age, pre-pregnancy BMI, and multiple gestations) | | | | | | | | | | | | | | | | |
| 2011-2013 | Ref | | Ref | | - | | Ref | | Ref | | - | | Ref | | Ref |  |
| 2014-2015 | **1.27**  **(1.05, 1.54)** | | 0  (0, INF) | | Ref | | 1.10  (0.65, 1.85) | | 1.32  (0.90, 1.92) | | Ref | | **1.31**  **(1.01, 1.72)** | | 1.30  (0.29, 5.80) |  |
| 2016-2018 | **1.25**  **(1.05, 1.49)** | | 1.28  (0.21, 7.72) | | 0.99  (0.62, 1.60) | | 0.77  (0.47, 1.27) | | 1.20  (0.85, 1.69) | | 0  (0, INF) | | **1.43**  **(1.12, 1.81)** | | 1.35  (0.35, 5.24) |  |
| 2019-2021 | **1.28**  **(1.07, 1.53)** | | 0.50  (0.04, 5.53) | | 1.09  (0.67, 1.78) | | 1.12  (0.71, 1.79) | | 0.99  (0.69, 1.42) | | 1.10  (0.10, 12.55) | | **1.44**  **(1.13, 1.83)** | | 1.45  (0.38, 5.50) |  |
| Also adjusted for co-morbidity indicators (assisted reproductive technology, pre-pregnancy diabetes, pre-pregnancy hypertension, gestational diabetes, preeclampsia/eclampsia, and high infant birth weight) | | | | | | | | | | | | | | | | |
| 2011-2013 | Ref | | Ref | | - | | Ref | | Ref | | - | | Ref | | Ref |  |
| 2014-2015 | **1.27**  **(1.05, 1.54)** | | 0  (0, INF) | | Ref | | 1.09  (0.65, 1.84) | | 1.31  (0.90, 1.90) | | Ref | | **1.31**  **(1.00, 1.71)** | | 1.31  (0.29, 5.88) |  |
| 2016-2018 | **1.23**  **(1.04, 1.47)** | | 1.27  (0.21, 7.66) | | 0.97  (0.60, 1.56) | | 0.76  (0.46, 1.25) | | 1.18  (0.83, 1.66) | | 0  (0, INF) | | **1.40**  **(1.10, 1.78)** | | 1.37  (0.35, 5.29) |  |
| 2019-2021 | **1.23**  **(1.03, 1.47)** | | 0.46  (0.04, 5.24) | | 1.03  (0.63, 1.67) | | 1.08  (0.68, 1.72) | | 0.96  (0.67, 1.38) | | 1.27  (0.11, 14.57) | | **1.38**  **(1.09, 1.76)** | | 1.44  (0.38, 5.45) |  |
| Also adjusted for obstetric practice factors (trial of labour, induction of labour, augmentation of labour) | | | | | | | | | | | | | | | |  |
| 2011-2013 | Ref | | Ref | | - | | Ref | | Ref | | - | | Ref | | Ref |  |
| 2014-2015 | **1.26 (1.04, 1.53)** | | 0  (0, INF) | | Ref | | 1.09 (0.65, 1.84) | | 1.30  (0.89, 1.90) | | Ref | | **1.31**  **(1.00, 1.70)** | | 1.27  (0.28, 5.70) |  |
| 2016-2018 | **1.23**  **(1.03, 1.46)** | | 1.22  (0.20, 7.51) | | 0.97  (0.60, 1.56) | | 0.76  (0.46, 1.25) | | 1.17  (0.83, 1.65) | | 0  (0, INF) | | **1.40**  **(1.10, 1.77)** | | 1.32  (0.34, 5.11) |  |
| 2019-2021 | **1.22**  **(1.02, 1.46)** | | 0.47  (0.04, 5.37) | | 1.03  (0.64, 1.69) | | 1.08  (0.67, 1.71) | | 0.94  (0.65, 1.35) | | 1.17  (0.10, 13.55) | | **1.38**  **(1.08, 1.76)** | | 1.34  (0.35, 5.13) |  |

Statistical significance was set at α < 0.05. Bolded text indicates statistical significance as per p-values (not shown). Sequential adjustment was performed by fitting a series of models in the order outlined above to quantify the contribution of additional groups of factors on hysterectomy trends over time.

AIAN, American Indian or Alaskan Native; NHOPI, Native Hawaiian or Other Pacific Islander; > 1 race, more than one race. All race/ethnicity categories were restricted to non-Hispanic individuals, except for those in the Hispanic group.

BMI; body mass index.
